# Supplementary material for: Implementing the NICE osteoarthritis guidelines: a mixed methods study and cluster randomised trial of a model osteoarthritis consultation in primary care - the Management of OsteoArthritis In Consultations (MOSAICS) study protocol
Source: Implement Sci. 2014 Aug 27;9:95. doi: 10.1186/s13012-014-0095-y (PMC4176866; doi:10.1186/s13012-014-0095-y)
Supplement: Additional file 1: — Resources provided. [file 13012_2014_95_MOESM1_ESM.docx]

### Additional file 1: Resources provided

Clinical and service support for the study will be provided by GP practices belonging to the Primary Care Research Network – Central England and North West. Research support costs are provided by Keele University via grants provided by the National Institute of Health Research (reference: RP-PG-0407-10386) and Arthritis Research UK (reference: 18139). All practices will be reimbursed for: screening a patient list for the population survey; OA template training; running the OA template in practice; clinical time and room hire for initial study set up meetings and for allowing PCRN access to run and download pseudo-anonymised medical record reviews. This reimbursement is a total sum to cover the projected costs and is not dependent upon variables such as recruitment, template use, or other performance factors. In addition, the intervention practices will receive backfill to allow GPs and Nurses to attend the MOAC training and to carry out MOAC consultations. Keele University have also agreed to support nurses who are interested in accrediting their MOAC 2 training through appropriate award-based modules at the University. All practices will be allocated a GP research facilitator (GPRF) to be their lead point of contact throughout the study. Members of the multidisciplinary team linked to the intervention practices will be invited to discussion workshops and will be offered reimbursed of their time. Control practices will also be offered training at the end of the study.

A memorandum of agreement and a service level agreement (SLA) will be written for each practice. A lead GP at each practice will sign these agreements as well as a signatory from the relevant PCRN, Keele University and local R&D managers. These documents will state the responsibility of each party involved, and will attribute and detail reimbursement for all aspects of the study.
