# Supplementary material for: Implementing the NICE osteoarthritis guidelines: a mixed methods study and cluster randomised trial of a model osteoarthritis consultation in primary care - the Management of OsteoArthritis In Consultations (MOSAICS) study protocol
Source: Implement Sci. 2014 Aug 27;9:95. doi: 10.1186/s13012-014-0095-y (PMC4176866; doi:10.1186/s13012-014-0095-y)
Supplement: Additional file 2: — Consent form. [file 13012_2014_95_MOESM2_ESM.docx]

**Section E. Continuing to help with this study**

**Thank you very much for completing this questionnaire.**

Please ensure that you have read the enclosed information sheet that explains about the study.

**Please read and complete the following consent form, and then sign below.**

# Consent Form

I confirm that I have read and understood the study information sheet and am willing to take part in the study.

I understand that I can withdraw from the study at any time, and that this will not affect the care I receive in any way.

| **Please answer each statement by putting a cross in the box on on each line** | Yes | No |  |  |
| --- | --- | --- | --- | --- |
| I give my permission for my medical records to be reviewed …… | □ | □ |  |  |
| I am happy to be contacted again (this does not mean that you must take part in future – you are just agreeing to be contacted again)…………………………………………………………………. | □ | □ |  |  |
| I understand that relevant sections of anonymised research data collected about me during the study may be looked at by individuals from regulatory authorities or from the NHS Trust, as part of monitoring of this research project. I give permission for these individuals to have access to my data…………………. | □ | □ |  |  |

Signed: Date:

Please print your name and address:

Telephone number:

**Even if you would prefer us not to review your medical records or contact you again about the study, the answers you have given in this questionnaire will still be very important to us.**

**Please return your questionnaire in the pre paid envelope provided**

**(no stamp needed)**

### **Thank you for your help with this research study**
