# Supplementary material for: Implementing the NICE osteoarthritis guidelines: a mixed methods study and cluster randomised trial of a model osteoarthritis consultation in primary care - the Management of OsteoArthritis In Consultations (MOSAICS) study protocol
Source: Implement Sci. 2014 Aug 27;9:95. doi: 10.1186/s13012-014-0095-y (PMC4176866; doi:10.1186/s13012-014-0095-y)
Supplement: Additional file 3: — The MOAC intervention. [file 13012_2014_95_MOESM3_ESM.docx]

**The MOAC intervention**

*MOAC 1: Initial consultation with the GP*

In intervention practices, when a patient consults with peripheral joint pain, the GP will assess and diagnose the problem and if a diagnosis of OA is made will, in appropriate language and tailored to the patient's level of understanding and individual circumstances, give an explanation of OA and hand out the OA guidebook, offer support for self-management, promote the NICE core treatments, and explain the next steps – to read the guidebook and start to put into practice the self-management strategies it outlines, and a follow-up appointment with the practice nurse (MOAC 2).

*MOAC 2: Follow-up consultation with the practice nurse*

MOAC 2 constitutes a new service for OA patients. The timing of the first appointment with the nurse is likely to be two to four weeks after the initial GP consultation, to give patients time to read the guidebook and start their self-management strategies. The practice nurse will: use the guidebook as a resource to answer questions and clarify issues; review the self-management plan; negotiate and agree appropriate goals with every patient; discuss the need for pain relief and opportunities for healthy eating, physical activity and exercise as appropriate. The timing of up to three follow-up visits with the nurse will be agreed between the patient and the practice nurse, but will be scheduled in a flexible manner to provide most appropriate support for the patient’s self-management. The practice nurse consultations will be supported by a MOAC 2 CRF and a nurse toolkit. The CRFs will be audited for completeness and adherence to protocol.

*MOAC 3: Dissemination to other health care professionals*

The broader multidisciplinary team working with the intervention practices will be identified by the GPs and practices nurses. They will be given a copy of the guidebook and will be informed about the objectives of the study by the study team. Patients having received the model OA consultation will then have the opportunity for additional support during any opportunistic review by the multidisciplinary team. New patients identified opportunistically can be referred to MOAC 1 by the multidisciplinary team.
