# Supplementary material for: Implementing the NICE osteoarthritis guidelines: a mixed methods study and cluster randomised trial of a model osteoarthritis consultation in primary care - the Management of OsteoArthritis In Consultations (MOSAICS) study protocol
Source: Implement Sci. 2014 Aug 27;9:95. doi: 10.1186/s13012-014-0095-y (PMC4176866; doi:10.1186/s13012-014-0095-y)
Supplement: Additional file 4: — List of Read codes. [file 13012_2014_95_MOESM4_ESM.docx]

| **Read codes/terms from EMIS system** | **Category** |
| --- | --- |
| ***National codes*** |  |
| N05  (plus all daughter codes) | Osteoarthritis  Osteoarthritis+allied disord |
| NO6Z3  NO6Z4  NO6Z5  NO6Z6  NO6Z7 | Arthropathy NOS-forearm  Wrist arthritis NOS  Arthropathy NOS of the hand  Arthropathy NOS-hand  Hand arthritis NOS  Hip arthritis NOS  Knee arthritis NOS  Ankle arthritis NOS  Foot arthritis NOS |
| NO94  NO940  NO943  NO944  NO945  NO946  NO947  NO94F  NO94G  NO94H  NO94K  NO94M  NO94P  NO94T  NO94W | Ache in joint  Pain in joint – arthralgia  Arthralgia - site unspecified  Arthralgia of unspecified site  Arthralgia - forearm  Wrist joint pain  Arthralgia - hand  Arthralgia of the hand  Hand joint pain  Arthralgia - pelvic/thigh  Coxalgia  Hip joint pain  Arthralgia - lower leg  Arthralgia of the lower leg  Knee joint pain  Ankle joint pain  Ankle/foot joint pain  Arthralgia - ankle/foot  Arthralgia of the ankle and foot  Arthralgia of wrist  Arthralgia of MCP joint  Arthralgia of PIP joint of finger  Arthralgia of hip  Hip pain  Arthralgia of knee  Arthralgia of ankle  Arthralgia of 1st MTP joint  Anterior knee pain |
| N2450  N2451 | Finger pain  Hand pain  Thumb pain  Foot pain  Toe pain |
| 1M10  1M11  1M13 | Knee pain  Foot pain  Ankle pain |
| ***Local codes*** |  |
| EGTON279  MAWBYKN1  MUNNUKN1 | Painful Right Knee  Knee Pain  Knee pain |

Additional File 4
