# Supplementary material for: Implementing the NICE osteoarthritis guidelines: a mixed methods study and cluster randomised trial of a model osteoarthritis consultation in primary care - the Management of OsteoArthritis In Consultations (MOSAICS) study protocol
Source: Implement Sci. 2014 Aug 27;9:95. doi: 10.1186/s13012-014-0095-y (PMC4176866; doi:10.1186/s13012-014-0095-y)
Supplement: Additional file 5: — Sensitivity analyses. [file 13012_2014_95_MOESM5_ESM.docx]

**Sensitivity analyses**

*Sensitivity analysis* 1 will be carried out using linear mixed modelling with GP Practitioner replacing GP Practice at level 3 (this is because a limitation of the main study is the small number of GP Practices – 4 per arm – which questions the reliability of the underlying variability at this level). The CONSORT guidelines for cluster trials indicate that the minimum number per arm at the highest level is 4 for purposes of validity. For this reason, our proposed sensitivity analysis will assess the robustness of the estimates using GP Practitioners at this level. GP Practitioners are likely to be the main contributors to the variation between GP Practices and may therefore be considered to be reasonable substitutes for the hierarchical model (and give a legitimate way of assessing the robustness of findings).

*Sensitivity analysis* 2 will be carried out at cluster level using permutation test; this is a useful non-parametric test that can be used for evaluating small numbers of clusters (Hayes and Moulton, 2009).

**Reference**

Hayes RJ, Moulton LH. Cluster Randomised Trials. Boca Raton, FL: Chapman & Hall/CRC; 2009.
