# Supplementary material for: Implementing the NICE osteoarthritis guidelines: a mixed methods study and cluster randomised trial of a model osteoarthritis consultation in primary care - the Management of OsteoArthritis In Consultations (MOSAICS) study protocol
Source: Implement Sci. 2014 Aug 27;9:95. doi: 10.1186/s13012-014-0095-y (PMC4176866; doi:10.1186/s13012-014-0095-y)
Supplement: Additional file 6: — Health economics analysis plan. [file 13012_2014_95_MOESM6_ESM.docx]

**Health Economics analysis plan**

***Overview***

The economic evaluation to be conducted alongside the MOSAICS trial will determine the cost-effectiveness of the template and model OA consultation (MOAC) in comparison to template alone in patients who consult with osteoarthritis (OA).

A cost-consequence analysis will initially be reported, describing all the important results relating to costs and consequences (clinical outcomes, EQ-5D, SF-6D, ICECAP-A). Subsequently, an incremental cost-utility analysis will also be undertaken using patient responses to the EQ-5D questionnaire (at ‘baseline’, 3, 6 and 12 months) to calculate the cost per additional quality-adjusted life year (QALY) gained. The base-case analysis will be conducted from a health service perspective.

***Costs***

Information on resource use and time off work due to joint problems will be collected from the postal MOSAICS questionnaires completed by patients at 6 months and 12 months. Health sector costs will include primary and secondary care contacts, investigations, medication and contacts with other health care professionals such as physiotherapists and occupational therapists (both through the NHS and private).

In order to obtain the cost of the MOAC intervention, information on the resources used to deliver the intervention will be obtained mainly through consultations with the study coordinators. Unit costs will then be applied to the resource use items. Since all practices received the template, a zero cost will be assigned to this intervention. Questions on patients’ personal expenditure will concentrate on private health care use and over-the-counter treatments. Questions on time off work and occupation will provide information required to calculate productivity losses (at 6 and 12 months).

Resource use will be multiplied by unit costs obtained from standard sources and health care providers (Curtis, 2012; BNF 2013; NHS reference costs 2012/13). Due to the lack of nationally representative unit cost estimates for private health care, this care will be costed as the NHS equivalent in the base-case. Patient reported costs for over-the-counter treatments will be used.

Productivity costs will be calculated using data collected on employment status at every time point and days off work due to their health. For those in paid employment, information on occupation, further details of typical work activities and the nature of their employment (full time or part time) will be requested. The average wage for each respondent will be identified using UK Standard Occupational Classification coding and annual earnings data for each job type. The analysis will use the human capital approach, and the self-reported days of absence will be multiplied by the respondent-specific wage rate. The human capital approach assumes that the value of lost work is equal to the amount of resources an individual would have been paid to do that work, and values productivity losses as a result of morbidity (or mortality) by measuring time lost from work and multiplying this with the gross wage of the person (Sculpher 2001).

***Outcomes***

Quality of life measures: All patients will be asked to complete the 3-level version of the EuroQoL-5D (EQ-5D) questionnaire (Rabin and de Charro 2001) at ‘baseline’, 3 months, 6 months and 12 months in order that quality-adjusted life years (QALYs) over the 12 month time period can be calculated for each study participant, using the area under the curve method (Matthews et al. 1990). Imbalances in baseline utility (EQ-5D) scores between the MOAC and template only groups will be controlled for using a regression approach (Manca et al. 2005). The QALY combines information on health-related quality of life and survival. The SF-12 will also be included as a measure of generic health-related quality of life, thus allowing an alternative method of calculating QALYs using the SF-6D in a sensitivity analysis. The ICECAP-A will also be included in questionnaires at all time points to measure broader aspects of quality of life.

***Data analysis***

The health economic analysis will determine the cost-utility of the template and model OA consultation (MOAC) in comparison to template alone. A cost-consequence analysis will initially be reported, describing all the important results relating to costs and consequences. (clinical outcomes, EQ-5D, SF-6D, ICECAP-A). Subsequently, an incremental cost-utility analysis will also be undertaken using patient responses to the EQ-5D questionnaire, to calculate the cost per additional QALY gained and incremental net benefits. This will form the base case and will adopt a National Health Service (NHS) and personal social services (PSS) perspective.

A broader costing perspective will be considered in a sensitivity analysis, taking into account NHS/PSS costs, patients’ personal expenditure and costs associated with work loss. The data for costs is likely to have a skewed distribution therefore the plan is to explore the nature of the distribution of costs. If the data are not normally distributed, the non-parametric comparison of means (e.g. bootstrapping) will be undertaken (Briggs and Gray 1999). Multiple imputation techniques (Rubin, 1987) will be used to deal with missing quality of life scores and resource use data, ensuring that all eligible trial participants are included in the base case economic evaluation.

Although standard methods have been developed for the analysis of clinical outcomes in cluster randomised trials, development of methods for conducting cost-effectiveness analyses alongside this type of trial has received relatively little attention in the literature. However, methods are required to address clustering in both costs and outcomes, and to recognise correlation between individual- and cluster-level costs and outcomes. Methods currently suggested in the health economics literature are multilevel models (MLM) and the 2-stage non-parametric bootstrap (TSB) (Gomes, 2012). However, it has been shown that MLM performs better. Therefore, for the base case scenario, MLM will be used to estimate differential costs, differential QALYs and incremental net benefits. The analysis will also allow us to control for covariates. Uncertainty will be explored through the use of cost-effectiveness acceptability curves (CEACs); these plot the probability that the intervention is cost-effective against threshold values for cost-effectiveness (Van Hout et al. 1994).

The robustness of the results will be explored using sensitivity analysis. This will explore uncertainties in the trial based data itself, the methods employed to analyse the data (for example, an available case analysis as an alternative to using an imputed data set) and the generalisability of the results to other settings.

**References**

Briggs AH, Gray AM. **Handling uncertainty when performing economic evaluation of health care interventions**. *Health Technology Assessment* 1999, **3**: 1-134.

BMJ Group. *British National Formulary (BNF) 65*. London: BMJ Group and RPS Publishing. 2013.

Curtis L. *Unit Costs of Health and Social Care 2012*. Personal Social Services Research Unit. University of Kent, Canterbury: 2012.

Department of Health NHS Reference Costs 2012/13 https://www.gov.uk/government/publications/nhs-reference-costs-financial-year-2011-to-2012

Gomes M, Ng E, Grieve R, Nixon R, Carpenter J, Thomson S. **Developing appropriate methods for cost-effectiveness analysis of cluster randomised trials.** *Medical Decision Making* 2012, **32**: 350-361.

Manca A, Hawkins N, Sculpher MJ. **Estimating mean QALYs in trial-based cost-effectiveness analysis: the importance of controlling for baseline utility.** *Health Econ* 2005, **14**: 487–496.

Matthews JN, Altman DG, Campbell MJ, Royston P. **Analysis of serial measurements in medical research.** *British Medical Journal* 1990, **300**: 230-235

Rabin R, de Charro F. EQ-5D: **A measure of health status from the EuroQol Group.** *Annals of Medicine* 2001, **33**: 337-343.

Rubin DB. *Multiple imputation for nonresponse in surveys*. New York, USA: John Willey & Sons; 1987.

Sculpher M. *The role and estimation of productivity costs in economic evaluation*; in: Drummond MF, McGuire A (eds): *Economic evaluation in health care: merging theory with practice*. Oxford: Oxford University Press; 2001.

Van Hou BA, AI MJ, Gordon GS, Rutten FF. Costs, effects and C/E ratios alongside a clinical trial. *Health Econ* 1994, **3**: 309-319.
